# Effects of Resisted Versus Non-Resisted Sprint Training on Countermovement Jump and Sprint Force–Velocity Profile in Youth Footballers: A Randomised Controlled Trial

**DOI:** 10.3390/sports14070258

**Published:** 2026-06-23

**Authors:** Tomas Ulloa-Guerrero, Juan S. Ruiz, Renato Rodríguez, Rafael Tadeo-Herazo, Sergio Lopez-Betancourt, Hermin Palacio-Bedoya, Samuel Gaviria-Alzate, Andrés Rojas-Jaramillo

**Affiliations:** 1 AFIS, Universidad de Antioquia, Medellín 050001, Colombia; tomasulloapf@gmail.com (T.U.-G.); juansebas0107@gmail.com (J.S.R.); renato130301@gmail.com (R.R.); rafael.herazo@udea.edu.co (R.T.-H.); alexanderbedoya333@gmail.com (H.P.-B.); 2 Universidad Pedagogica y Tecnologica de Colombia, Medellín 050001, Colombia; sergio.lopezbetancourt@uptc.edu.co; 3 Faculty of Education and Social Science, Tecnológico de Antioquia, Institución Universitaria, Medellín 050001, Colombia; samuel.gaviria@tdea.edu.co; 4 Research Division, Dynamical Business & Science Society—DBSS International SAS, Bogotá 110311, Colombia

**Keywords:** resisted sprinting, force–velocity profile, horizontal power, RFPeak, youth football, acceleration, maximal velocity

## Abstract

Background: In youth football, sprint performance depends on the capacity to produce and orient force horizontally during acceleration. Resisted sprinting may preferentially target the force end of the sprint force–velocity profile, whereas free sprinting may favour velocity-oriented adaptations. Purpose: To compare the effects of resisted versus non-resisted sprint training on sprint performance and sprint force–velocity variables in youth footballers, while monitoring countermovement jump (CMJ) as a secondary outcome. Methods: This parallel-group randomised controlled trial included 44 players from two age categories (U14, n = 21; Youth, n = 23). Within each category, players were randomly allocated to resisted sprint training (RST; U14 n = 11, Youth n = 12) or non-resisted sprint training (NRST; U14 n = 10, Youth n = 11). Both groups completed two supervised sessions per week for six weeks. Outcomes were CMJ and sprint-derived variables including maximal theoretical horizontal force (F0), maximal theoretical velocity (V0), maximal power (Pmax), measured maximal sprint velocity (Vmax), peak ratio of horizontal force (RFpeak), decrease in RF with increasing velocity (DRF), and force–velocity slope (FV). Results: CMJ remained essentially unchanged in both age categories. Sprint performance improved over time, with the pattern of adaptation generally favouring RST for force-oriented sprint mechanical variables (F0, Pmax and RFpeak), whereas improvements in Vmax were observed in both groups. In the Youth category, the FV slope differed between groups post-test (*p* = 0.002). Overall, resisted sprint training tended to produce larger improvements in acceleration-oriented mechanical qualities, while non-resisted sprint training was associated with more velocity-oriented adaptations. Conclusions: Low-volume resisted sprint training using a sled load of ~20% body mass was associated with more favourable adaptations in force-oriented sprint mechanical variables, whereas non-resisted sprint training tended to favour velocity-oriented characteristics. CMJ performance remained unchanged in both groups. These findings should be interpreted cautiously given the small age-stratified subgroup sizes and the single-club nature of the study. Trial registration: This study was retrospectively registered at ClinicalTrials.gov (NCT07418892).

## 1. Introduction

Sprinting is a decisive action in football because it underpins separation from opponents, runs into space, and ball contests [1,2,3]. In youth cohorts, the ability to generate horizontal force and express speed efficiently is a key component of athletic development and match performance [4,5,6]. Sprint performance is usually described through acceleration and maximal-velocity phases, each placing different mechanical demands on the athlete [7,8]. Within this framework, coaches commonly use resisted sprinting (e.g., sled towing) and non-resisted sprinting to bias the training stimulus towards specific segments of the sprint force–velocity profile [9,10,11]. Although football is characterised by intermittent and multidirectional movement patterns, including accelerations, decelerations, and changes in direction, straight-line sprinting remains a decisive action during critical match events. Faude et al. [1] reported that straight sprinting was the most frequent action preceding goal-scoring situations in professional football, occurring more often than changes in direction, jumps, tackles, or technical actions immediately before goals and assists. Therefore, despite the complex movement demands of football, linear sprint performance remains a highly relevant physical quality for match success and warrants specific investigation.

Current evidence suggests that resisted sprint training can improve short-sprint performance, particularly when the load is selected to emphasise horizontal force production during acceleration. Previous research has shown that resisted sprint training, particularly using sled towing, can improve acceleration performance by increasing the horizontal force demands during the early steps of the sprint. Studies in team-sport athletes have reported improvements in short-sprint performance and sprint mechanical variables when external loads are used to emphasise force production during acceleration [9,10,11,12]. By contrast, free sprinting and longer high-speed exposures may be more relevant when the objective is to preserve sprint mechanics and develop velocity-oriented qualities [6,13,14]. Integrated programmes combining strength, plyometrics, and sprinting can also enhance related performance outcomes such as jumping and change-of-direction ability, although transfer depends on the vector and specificity of the stimulus [15,16,17,18].

Beyond sprint time alone, sprint force–velocity profiling allows practitioners to distinguish the mechanical contributors to performance—maximal theoretical horizontal force (F0), maximal theoretical velocity (V0), maximal power (Pmax), and variables describing the effectiveness of force application, such as RFpeak and DRF [7,8,19]. This framework provides a more mechanistic basis for load prescription and monitoring than time-based outcomes alone and is increasingly used to individualise sprint training in team-sport athletes [7,20,21].

Previous resisted sprint training studies have used a wide range of methodological approaches. Sprint distances have typically ranged from 10 to 30 m, intervention durations from 4 to 8 weeks, and sled loads from light resistance (<10% body mass) to heavy and very-heavy loading strategies exceeding 20% of body mass [11,12,13,14,21,22]. Furthermore, recent systematic reviews have highlighted substantial heterogeneity in training prescription, participant characteristics and outcome measures, making direct comparisons between studies difficult and limiting practical recommendations for youth football populations [9,10,11].

However, evidence in youth football remains fragmented. Studies frequently combine heterogeneous sled loads, brief interventions, or mixed training contents, and relatively few directly compare resisted and non-resisted sprint training under comparable exposure while reporting the full set of sprint mechanical outcomes [6,10,22,23]. In addition, maturation-related differences may influence how young players respond to force- or velocity-oriented sprint stimuli [4,5,24].

Therefore, the aim of this randomised controlled trial was to compare six weeks of resisted sprint training using a sled load of 20% body mass with matched non-resisted sprint training in U14 and Youth footballers. We hypothesised that resisted sprint training would produce larger improvements in F0, Pmax, and RFpeak, whereas non-resisted sprinting would be more favourable for velocity-oriented adaptations, with minimal change in CMJ.

By contrasting two ecologically feasible sprint prescriptions within routine academy practice, the study seeks to provide coaches with clearer guidance on when to prioritise resisted or free sprinting according to the athlete’s mechanical profile and training objective.

## 2. Materials and Methods

### 2.1. Study Design and Setting

A parallel-group randomised controlled trial was conducted to compare the effects of resisted and non-resisted sprint training across a six-week intervention period [25]. The study was performed within the normal training routine of a local youth academy competing on the same third-generation artificial turf surface throughout the intervention.

**Figure 1:**
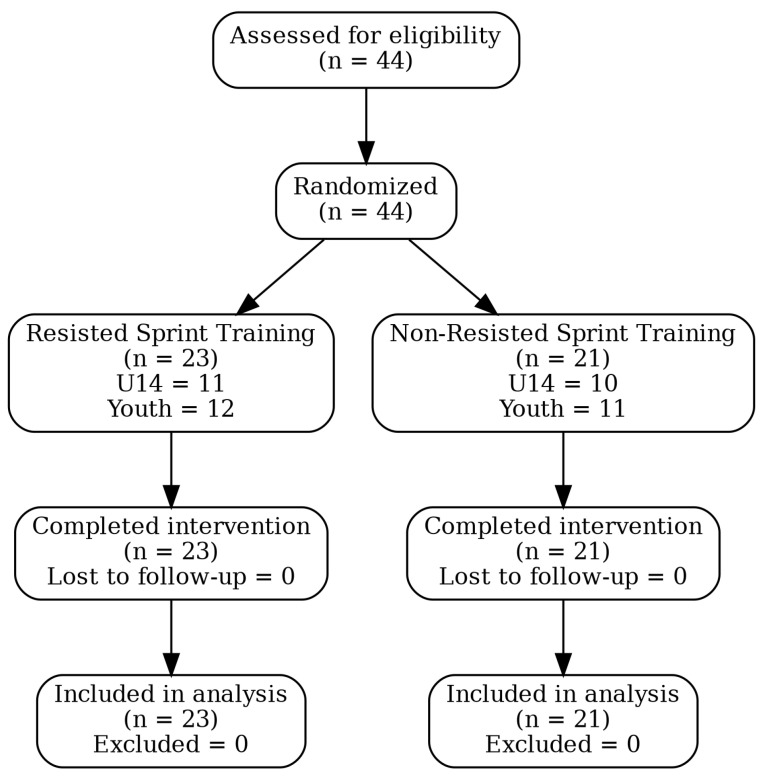
CONSORT-style flow diagram of screening, randomisation, follow-up, and analysis. Within-category allocation was U14: resisted n = 11, NRST n = 10; Youth: resisted n = 12, NRST n = 11. All randomised players completed follow-up and were included in the final analysis.

Players were first stratified by age category (U14 and Youth) and then randomly allocated within each stratum to resisted sprint training or non-resisted sprint training using computer-generated numbers prepared by a researcher not involved in training delivery [26]. The final analysed allocation was U14 resisted n = 11, U14 NRST n = 10, Youth resisted n = 12, and Youth NRST n = 11 (Figure 1).

The intervention was embedded at the start of the habitual training session twice per week for six consecutive weeks under comparable environmental and scheduling conditions for both groups. Participant flow and final analysed allocation are shown in Figure 1. The trial was retrospectively registered at ClinicalTrials.gov (NCT07418892). The registration was completed after the start of participant enrolment and data collection; therefore, the study should be considered retrospectively registered.

### 2.2. Participants

Forty-four outfield players from two academy squads were enrolled (U14: n = 21; Youth: n = 23). All participants trained more than three times per week and competed regularly in a regional youth league, with some players also competing at national or international level. According to McKay’s Participant Classification Framework [27], the cohort was predominantly Tier 2–3, with a subset classified as Tier 3–4 based on training history and competitive level. Baseline anthropometric characteristics by age category are shown in Table 1.

### 2.3. Eligibility Criteria

Inclusion: Registered U14 or Youth players; ≥1 year of organised football experience; current health-system affiliation; provided informed assent (and parental/guardian consent for minors).

Exclusion: Current osteomuscular complaint; injury incurred during the intervention; ongoing rehabilitation; logistical constraints preventing participation in the sessional protocol. The U15 squad was excluded due to venue unavailability for the full intervention dose.

### 2.4. Sampling and Randomisation

Within each category (U14; Youth), players were assigned to the experimental or control group using simple randomisation (computer-generated random numbers) [26]. To reduce selection bias, the randomisation list was prepared by a researcher not involved in training delivery, and group lists were released to coaches only after assignment.

Recruitment was population-based within the accessible sampling frame: all eligible players from the two intact academy squads who were available during the study period were invited to participate and were enrolled. In line with contemporary guidance on sample-size justification, this design reflects the use of the available population in an applied sport setting rather than a formal power-based recruitment target [28]. Consequently, the trial should be interpreted as an exploratory club-based randomised study aimed at estimating the direction and practical magnitude of effects, not as a definitive trial designed for broad generalisation.

### 2.5. Ethics

The study protocol was approved by the Research Ethics Committee of the Universidad de Antioquia (Approval No. CE-042024-6-2024; 17 April 2024). Procedures complied with the Declaration of Helsinki and with Colombian Resolution 8430 of 1993 governing research involving human participants [29]. Written assent was obtained from the players and written consent from parents or legal guardians when applicable. Personal data were coded and stored in a password-protected file accessible only to the research team.

### 2.6. Outcomes

Primary outcomes (sprint mechanics over 30 m)

Horizontal sprint force–velocity–power profile variables derived from split-time video analysis were the primary outcomes: maximal theoretical horizontal force (F0, N·kg^−1^), maximal theoretical running velocity (V0, m·s^−1^), maximal horizontal power (Pmax, W·kg^−1^), measured maximal sprint velocity (Vmax, m·s^−1^), peak ratio of horizontal force (RFpeak, %), decrease in RF with increasing velocity (DRF, %·m^−1^), force–velocity slope (FV), and best 30 m sprint time (s). Variable definitions followed the framework proposed by Morin and Samozino [7,8].

Secondary outcomes

Countermovement jump (CMJ), height (cm), stature (m), body mass (kg) and derived BMI (kg·m^−2^).

### 2.7. Instruments and Validity

CMJ was assessed with a Chronojump open-source contact platform, which has shown acceptable validity and reliability for jump-height assessment in sport settings. Sprint profiling was obtained with the MySprint v 2.5 iOS application using 240 fps video to derive split times every 5 m and estimate force–velocity–power outputs from the Samozino inverse-dynamics model; MySprint has shown good concurrent validity and reliability against reference systems [8,30].

### 2.8. Procedures

#### 2.8.1. Familiarisation and Pilot Work

Two pilot sessions were undertaken to rehearse logistics and refine protocols: (i) 25 September 2024, CMJ procedures with both categories; (ii) 27 September 2024, 30 m sprint profiling with all three squads. These were conducted at high altitude (~1500 m above sea level) under temperate conditions; ambient temperature and relative humidity were recorded prior to testing.

#### 2.8.2. Testing Schedule

Pre- and post-intervention assessments comprised anthropometrics, CMJ height and 30 m sprint profiling. All assessments were performed on the same day and at the same time of day for each category, with ≥72 h since the last match to minimise fatigue carry-over.

#### 2.8.3. Standardised Warm-Up

For CMJ: 5 min easy running and joint mobility, then 10 practice CMJs using the test technique.

For sprint profiling: 5 min easy running and joint mobility; five submaximal 20 m runs without external load; three 20 m maximal efforts.

#### 2.8.4. CMJ Protocol

**Figure 2:**
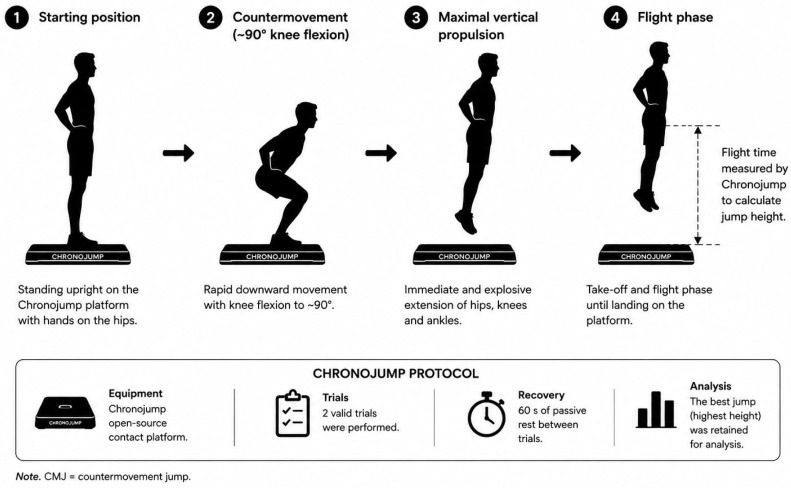
Countermovement jump (CMJ) assessment procedure.

Participants stood upright with hands on hips throughout. After a rapid countermovement to ~90° knee flexion (sagittal-plane verification), they performed a maximal vertical jump. The best of two valid attempts was recorded (inter-attempt rest ≥ 60 s) (Figure 2).

#### 2.8.5. Sprint Force–Velocity Profiling Protocol

**Figure 3:**
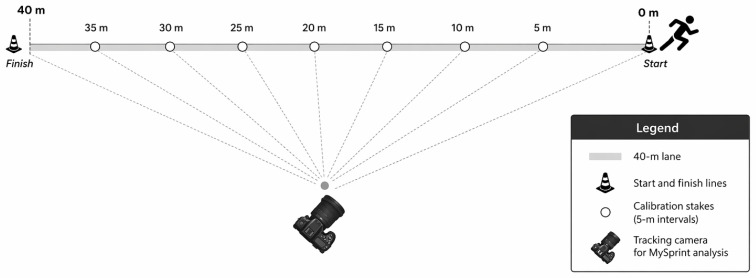
Plan-view schematic of the 30 m sprint set-up showing the 40 m lane, 5 m split intervals, lateral calibration stakes, and the position of the tracking camera used for MySprint analysis.

A 40 m lane was laid out to capture 30 m of timed acceleration while ensuring athletes did not decelerate before the 30 m line. Split markers were positioned every 5 m (0, 5, 10, 15, 20, 25, and 30 m), and the spatial arrangement of the lane, calibration stakes, and camera track is shown in Figure 3.

To achieve accurate parallax and split-time detection in MySprint, stake positions were marked at lateral offsets consistent with the app’s calibration guidance (0 m = 1.43 m; 5 m = 5.95 m; 10 m = 10.48 m; 15 m = 15.00 m; 20 m = 19.53 m; 25 m = 24.05 m; 30 m = 28.58 m). Each athlete completed two maximal runs from a standing start; the best trial was analysed.

Start criteria: After the recording started, athletes self-initiated the sprint when ready, without rocking, with one foot on or behind the start line and remaining within the coned lane. Time zero was the first forward propulsive movement detected on video.

Video files were imported into MySprint to derive split times and compute mechanical outputs from the Samozino model (step-averaged horizontal ground-reaction forces in the sagittal plane estimated from body mass and spatiotemporal data).

### 2.9. Interventions

#### 2.9.1. Experimental Group—Resisted Sprints

Twenty-metre maximal sprints towing a sled loaded to 20% of individual body mass (sled tare mass 3 kg; plates added to reach the target). Dose: 3 sets × 5 repetitions; inter-rep rest: 45 s; inter-set rest: 3 min.

#### 2.9.2. Control Group—Non-Resisted Sprints

Twenty-metre maximal sprints without external resistance. Dose and rests identical to the experimental group.

The sprint-training intervention was performed twice weekly (Tuesdays and Thursdays) for six weeks, with approximately 48 h between sessions. To minimise the effects of accumulated fatigue, all sprint-training sessions were completed at the beginning of the football training session before any technical or tactical activities were undertaken.

Both groups participated in the same technical, tactical, and conditioning activities prescribed by the coaching staff. The sprint-training intervention was the only planned difference between groups.

### 2.10. Common Features

Two supervised sessions per week for six consecutive weeks; interventions were placed at the start of the squad training session; identical surface (third-generation artificial turf); coned 20 m lane plus 5 m runoff to discourage pre-30 m braking. No additional lower-limb strength or speed training beyond the head coach’s standard plan was introduced during the intervention period. The sprint-training sessions were conducted every Tuesday and Thursday throughout the intervention period, providing approximately 48 h between exposures. No progression in sprint distance, volume, or external load was implemented, and all training variables remained constant throughout the six-week intervention.

### 2.11. Bias Control and Data Management

Randomisation was stratified by category to balance maturation and training status [31]. Players were not told which condition was considered “experimental” versus “control”, although full participant blinding to the presence of a sled is not feasible. Video analysts and the statistician were blinded to group codes. Athletes were identified by coded numbers in the dataset; consent forms were checked for completeness; data were entered into a locked Excel file by two investigators (double-entry verification). Complete participant blinding was not feasible due to the visible nature of the sled-resisted intervention.

### 2.12. Adverse-Event Monitoring and First-Aid Procedures

Before each session, players reported any osteomuscular complaints, pre-session meals and previous night’s sleep. Standard first-aid coverage was present at training. The RICE protocol was available for minor contusions.

### 2.13. Statistical Analysis

All analyses were conducted in Jamovi (v2.3.28). Distributions were screened using the Shapiro–Wilk test and visual inspection. Descriptive statistics are reported as mean ± SD. The primary analytical focus was the group × time comparison for CMJ and sprint mechanical variables using mixed repeated-measures models, with Greenhouse–Geisser correction when sphericity was violated [32]. Because subgroup sizes were modest, emphasis was placed on the direction and magnitude of effects, alongside exact *p* values, 95% confidence intervals where appropriate, and Cohen’s d effect sizes interpreted using conventional thresholds [33]. Missing outcome data were minimal and were handled by complete-case analysis.

## 3. Results

### 3.1. Participant Characteristics

Forty-four players completed the study and were included in the pooled analysis (RST n = 23; NRST n = 21). Age-stratified allocation was U14 RST n = 11, U14 NRST n = 10, Youth RST n = 12, and Youth NRST n = 11 (Figure 1). Baseline anthropometric characteristics by age category are presented in Table 1, and baseline training-group descriptives for the pooled sample are shown in Table 2.

Pre-test, most pooled outcomes were approximately normally distributed; FV and RFpeak were the only variables showing non-normality. At post-test, all pooled outcomes met the normality assumption.

In the pooled analysis (Table 3), CMJ showed trivial change in both groups. Sprint time improved in both groups, but the reduction was larger in RST (ES = −0.88) than in CON (ES = −0.35). Vmax and V0 increased in both groups, with no between-group post-test difference. By contrast, the force-oriented variables responded more specifically to the resisted stimulus: F0 and Pmax increased substantially in RST, whereas CON showed no meaningful improvement in either variable, resulting in significant post-test differences favouring RST (F0 *p* = 0.011; Pmax *p* = 0.010). RFpeak and the FV slope also improved in the resisted condition, whereas DRF showed only a small change in CON.

#### 3.1.1. Under-14 (U14)

In U14 players (Table 4), both groups reduced 30 m sprint time, and the between-group post-test difference was not significant. Nevertheless, the mechanical pattern differed between conditions: RST produced moderate improvement in F0 and a large improvement in Pmax, whereas NRST showed its clearest change in Vmax and little evidence of improvement in force-oriented variables. RFpeak also tended to improve more in RST, although the between-group contrast did not reach statistical significance.

#### 3.1.2. Youth

In Youth players (Table 5), the distinction between training modes was clearer. RST produced a large reduction in sprint time and substantial gains in F0, Pmax, and RFpeak, whereas NRST showed only a small change in sprint time, a decline in F0, and no meaningful change in Pmax. Although both groups increased Vmax and V0, post-test values favoured RST for F0 and RFpeak, and the FV slope differed significantly between groups (*p* = 0.002), indicating a more force-oriented adaptation after resisted training.

## 4. Discussion

The main contribution of this trial is that the two sprint prescriptions produced distinct mechanical adaptation patterns despite identical session frequency and nominal exposure. Resisted sprint training shifted performance towards the force-oriented end of the profile—especially F0, Pmax, and RFpeak—whereas non-resisted sprinting mainly preserved or modestly improved velocity-related qualities. This pattern is practically important because faster sprint times can arise from different mechanical routes, and the present data indicate that a sled load of ~20% body mass preferentially strengthened the acceleration-related route in these youth footballers [7,9,10,11].

This interpretation is mechanically coherent. Over the first metres of a sprint, performance depends on the athlete’s ability to generate and project large horizontal forces; therefore, increases in F0 and Pmax provide a plausible explanation for the larger sprint-time improvements observed after resisted training [7,19]. Previous sled-based studies and syntheses likewise show that moderate-to-heavy resisted sprinting is especially effective for enhancing acceleration mechanics and short-sprint performance when compared with non-resisted running alone [10,11,12,23].

By contrast, the improvements in Vmax and V0 observed in both groups—and particularly the relative response of the U14 controls—suggest that younger players may still benefit from repeated free-sprint exposures when the aim is to enhance the velocity end of the profile. This age-dependent pattern is consistent with work showing that maturation status influences whether sprint adaptations are expressed more strongly through force- or velocity-oriented variables [4,5,24,34]. In practical terms, younger or more velocity-deficient players may not require external resistance to improve their high-speed qualities, whereas more mature athletes may need a stronger force stimulus to meaningfully shift the profile.

The behaviour of RFpeak reinforces this interpretation. RFpeak reflects how effectively the athlete directs the resultant force horizontally during the most propulsive steps [19,35], and its improvement after resisted training suggests better mechanical alignment for acceleration. The fact that RFpeak declined in the Youth control group while increasing in the resisted group implies that sprint exposure alone did not provide the same stimulus for horizontal force orientation under the present training conditions. The small and inconsistent changes in DRF also suggest that the intervention affected the initial force-production ceiling more than the maintenance of force orientation as speed increased [36,37].

The absence of a meaningful CMJ response in either condition is also informative rather than disappointing. The intervention was brief, horizontally oriented, and embedded within the start of routine football practice; under such conditions, transfer to a vertically dominated task such as CMJ should not be assumed. This selective transfer is compatible with vector-specific adaptation principles and with previous work showing that improvements in sprint mechanics do not necessarily translate to vertical jump performance unless the programme explicitly includes a vertical or mixed-force stimulus [16,17,38].

From an applied perspective, the present dose appears feasible for academy settings: two weekly exposures, low total volume, and a moderate sled load were sufficient to elicit clear mechanical changes without obvious adverse effects. The practical implication is not that resisted sprinting is categorically superior, but that it is more appropriate when the performance problem is force-side constrained. Conversely, free sprinting remains useful when the coaching goal is to rehearse high-speed mechanics or support velocity development, particularly in younger players. Thus, the choice between resisted and non-resisted sprinting should be made against the athlete’s current force–velocity profile rather than by default [7,11,21].

These interpretations must nevertheless remain proportionate to the design. The trial was conducted in a single club, the age-stratified subgroups were small, and the intervention lasted only six weeks. Accordingly, the study is better viewed as mechanistically informative and practice-oriented than as definitive evidence that one method will generalise to all youth football populations.

Although football is characterised by multidirectional movement patterns involving accelerations, decelerations, and changes in direction, linear sprinting remains a decisive action during critical match situations. Faude et al. reported that straight sprinting was the most frequent action preceding goals and assists in professional football, highlighting its relevance to match performance. Therefore, the present intervention should not be interpreted as an attempt to replicate the full complexity of football-specific movement demands, but rather as a targeted strategy to improve the mechanical qualities underpinning acceleration and sprint performance. Nevertheless, future studies should investigate whether the adaptations observed following resisted sprint training transfer to more ecologically valid tasks, such as change-of-direction performance and repeated sprint ability.

Although resisted sprint training is primarily designed to enhance sprint-specific mechanical qualities, the absence of significant changes in CMJ performance should not be interpreted solely through the lens of horizontal force specificity. Sprinting involves substantial vertical force production, rapid stretch–shortening cycle utilisation, and high neuromuscular demands. Therefore, some transfer to vertical jump performance could theoretically be expected. Nevertheless, the relatively low training volume, short intervention duration, and the highly task-specific nature of the stimulus may have limited the development of adaptations sufficient to improve CMJ performance. This interpretation is consistent with the principle of specificity while recognising the shared neuromuscular determinants underpinning sprinting and jumping tasks.

## 5. Limitations of the Study and Future Research Directions

Several limitations should be acknowledged. First, although all available eligible players from the accessible academy population were included, stratification by age and training condition produced small subgroup sizes, which reduced precision for between-group comparisons and limits broader generalisation. Second, the intervention duration was sufficient to detect short-term mechanical adaptations but may have been too short to reveal slower neuromuscular or structural changes, including any clearer transfer to CMJ.

Third, external training load and fatigue were not controlled beyond the routine team schedule, so match demands or residual fatigue may have influenced variables such as DRF and the FV slope. Fourth, as in other field-based sprint-profiling studies, estimates depended on video-derived split times and model-based calculations; although these methods are validated, methodological heterogeneity across studies still complicates direct comparison of absolute values [8,30,39,40].

A further limitation is the absence of a direct assessment of biological maturation. Although participants were stratified and randomised within age categories, no maturity-specific indicators such as maturity offset, peak height velocity, Tanner stage, or skeletal age were collected. Consequently, it is not possible to determine the extent to which individual differences in biological maturation influenced the observed training responses. Future studies should incorporate validated maturation assessments to better understand the interaction between maturity status and sprint-training adaptations. Additionally, all participants were recruited from a single football academy. Consequently, the observed adaptations may have been influenced by club-specific factors such as training philosophy, coaching practices, training history, competitive level, and weekly training load. Therefore, caution is warranted when extrapolating the present findings to other youth football populations. Future multi-club studies are needed to determine the generalisability of these results across different developmental and training environments.

Future studies should test longer interventions, incorporate systematic monitoring of maturation and external load, and examine whether an explicitly profile-based allocation of resisted versus non-resisted sprinting enhances the individual response in youth footballers. Multi-club trials with larger samples would also help determine how robust the present mechanical patterns are across competitive standards and developmental stages. Finally, only male youth football players were included in the present investigation. Consequently, the findings cannot be directly generalised to female football populations, who may exhibit different neuromuscular characteristics and training responses. Future studies should examine the effects of resisted and non-resisted sprint training in female youth football players.

## 6. Practical Applications

From an applied standpoint, the results suggest that resisted and unresisted sprint training should not be viewed as interchangeable stimuli in youth football. Light resisted sprinting (~20% body mass) appears most relevant when the training aim is to improve acceleration-oriented mechanical qualities, particularly F0, Pmax, and RFpeak, whereas unresisted sprint exposure may be more suitable when the priority is to develop velocity-oriented qualities, especially in younger players. In practice, the value of these methods is likely to be maximised when training decisions are guided by the athlete’s sprint force–velocity profile rather than by a standardised programme applied uniformly across the squad. Because the present findings derive from a brief intervention and relatively small age-stratified groups, they should be interpreted as context-specific applied guidance rather than as definitive prescriptions for all youth football populations.

## 7. Conclusions

Both resisted and unresisted sprint training were associated with faster 30 m sprint times in youth footballers; however, resisted sprinting produced the more consistent improvements in acceleration-oriented mechanical variables, particularly F0, Pmax, and RFpeak, without meaningful changes in CMJ. Unresisted sprinting appeared to favour velocity-oriented adaptations to a greater extent, particularly in the younger stratum. Taken together, these findings suggest that sprint training in youth football may be more effective when the choice of resisted or unresisted exposure is aligned with the athlete’s sprint force–velocity profile and the specific mechanical quality targeted. Given the relatively small subgroup sizes and short intervention duration, these findings should be interpreted as applied evidence for similar developmental football contexts rather than as broadly generalisable effects.

## Figures and Tables

**Table 1:** Baseline anthropometric characteristics by age category (Youth n = 23; U14 n = 21).

	Weight	Height	Age	BMI
Youth	62.8 ± 4.87	1.73 ± 0.04	15.8 ± 0.4	20.9 ± 1.19
U14	51.3 ± 6.94	1.62 ± 0.06	13.7 ± 0.3	19.5 ± 1.89

**Table 2:** Baseline descriptive characteristics by training group in the pooled sample.

Group (Training Allocation)	Weight (kg)	Height (m)	BMI (kg·m^−2^)
Resisted sprint (RST; n = 23)	58.5 ± 8.07	1.68 ± 0.07	20.5 ± 1.69
Unresisted sprint (NRST; n = 21)	56.0 ± 8.47	1.67 ± 0.09	19.9 ± 1.70

Note: Age was category-defined and therefore not summarised at pooled group level. BMI = body mass index.

**Table 3:** Pre- to post-training changes by group in the pooled sample (RST n = 23; NRST n = 21).

Variable	RST (n = 23) Pre	Post	ES	NRST (n = 21) Pre	Post	ES	Between-Group *p* PRE	Between-Group *p* POST
CMJ (cm)	35.44 ± 4.95	35.41 ± 4.66	−0.006	33.68 ± 5.66	34.60 ± 4.63	0.162	0.279	0.565
Sprint (s)	5.07 ± 0.28	4.82 ± 0.27 ***	−0.88	5.06 ± 0.28	4.96 ± 0.24 ***	−0.35	0.965	0.071
Vmax (m·s^−1^)	7.52 ± 0.51	7.71 ± 0.49 ***	0.37	7.38 ± 0.60	7.68 ± 0.52 ***	0.50	0.408	0.882
F0 (N·kg^−1^)	7.23 ± 1.70	8.24 ± 1.52 ***	0.59	7.48 ± 1.32	7.13 ± 1.26	−0.26	0.598	0.011
V0 (m·s^−1^)	7.83 ± 0.58	8.00 ± 0.53 **	0.29	7.67 ± 0.68	8.01 ± 0.57 **	0.501	0.408	0.942
Pmax (W·kg^−1^)	14.02 ± 2.88	16.4 ± 2.86 ***	0.83	14.27 ± 2.45	14.24 ± 2.41	−0.01	0.759	0.010
DRF (%·m^−1^)	−0.09 ± 0.02	−0.09 ± 0.03	−0.16	−0.09 ± 0.02	−0.08 ± 0.02 *	0.47	0.499	0.203
FV slope	−54.87 ± 16.41	−60.51 ± 14.21 ***	−0.34	−51.04 ± 28.85	−49.72 ± 11.27 *	0.046	0.963	0.007
RFpeak (%)	47.74 ± 6.42	51.83 ± 5.31 ***	0.63	48.71 ± 4.82	48.00 ± 4.36	−0.14	0.823	0.004

Note: Within-group significance: * *p* < 0.05, ** *p* < 0.01, *** *p* < 0.001. ES = Cohen’s d. Abbreviations: CMJ = countermovement jump; Vmax = maximal sprint velocity; F0 = theoretical maximal horizontal force; V0 = theoretical maximal running velocity; Pmax = maximal power output; RFpeak = peak ratio of force; DRF = decrease in the ratio of force; FV slope = force–velocity slope.

**Table 4:** Pre- to post-training changes by group in U14 players (RST n = 11; CON n = 10; abbreviations as in Table 3).

Variable	RST (n = 11) Pre	Post	ES	NRST (n = 10) Pre	Post	ES	Between-Group *p* PRE	Between-Group *p* POST
CMJ (cm)	32.78 ± 3.11	32.85 ± 2.97	0.023	30.79 ± 4.03	31.71 ± 3.84	0.227	0.160	0.557
Sprint (s)	5.25 ± 0.21	4.99 ± 0.23 ***	−1.245	5.30 ± 0.12	5.15 ± 0.17 **	−1.348	0.699	0.203
Vmax (m·s^−1^)	7.29 ± 0.53	7.44 ± 0.44	0.282	7.05 ± 0.63	7.33 ± 0.41 *	0.452	0.181	0.409
F0 (N·kg^−1^)	6.81 ± 1.82	7.87 ± 1.73 **	0.580	6.93 ± 1.38	6.76 ± 0.99	−0.124	0.637	0.179
V0 (m·s^−1^)	7.63 ± 0.66	7.75 ± 0.53	0.185	7.36 ± 0.77	7.66 ± 0.47 *	0.388	0.160	0.523
Pmax (W·kg^−1^)	12.74 ± 2.50	15.08 ± 2.69 ***	0.935	12.55 ± 1.60	12.90 ± 1.62	0.225	0.973	0.116
DRF (%·m^−1^)	−0.09 ± 0.03	−0.09 ± 0.03	−0.016	−0.09 ± 0.02	−0.08 ± 0.01	0.338	0.396	0.913
FV slope	−47.21 ± 15.45	−53.26 ± 13.87 ***	−0.391	−37.98 ± 36.49	−44.96 ± 11.12	−0.191	0.922	0.179
RFpeak (%)	45.55 ± 7.08	50.00 ± 6.10	0.630	46.00 ± 4.76	46.50 ± 3.89	0.105	0.680	0.280

Note: Within-group significance: * *p* < 0.05, ** *p* < 0.01, *** *p* < 0.001. ES = Cohen’s d.

**Table 5:** Pre- to post-training changes by group in Youth players (RST n = 12; CON n = 11; abbreviations as in Table 3).

Variable	RST (n = 12) Pre	Post	ES	NRST (n = 11) Pre	Post	ES	Between-Group *p* PRE	Between-Group *p* POST
CMJ (cm)	37.87 ± 5.17	37.75 ± 4.78	−0.02	36.31 ± 5.79	37.22 ± 3.70	0.158	0.566	0.67
Sprint (s)	4.90 ± 0.22	4.66 ± 0.20 ***	−1.05	4.84 ± 0.19	4.80 ± 0.16	−0.247	0.508	0.19
Vmax (m·s^−1^)	7.73 ± 0.40	7.95 ± 0.40 **	0.56	7.68 ± 0.40	8.00 ± 0.39 *	0.805	0.672	0.86
F0 (N·kg^−1^)	7.62 ± 1.57	8.59 ± 1.28 ***	0.62	7.98 ± 1.09	7.46 ± 1.42 *	−0.477	0.112	0.050
V0 (m·s^−1^)	8.01 ± 0.45	8.22 ± 0.44 *	0.47	7.95 ± 0.45	8.33 ± 0.46 *	0.836	0.636	0.70
Pmax (W·kg^−1^)	15.19 ± 2.78	17.63 ± 2.53 ***	0.87	15.83 ± 2.01	15.45 ± 2.42	−0.189	0.215	0.06
DRF (%·m^−1^)	−0.09 ± 0.02	−0.10 ± 0.02 *	−0.34	−0.09 ± 0.02	−0.08 ± 0.02 *	0.676	0.117	0.08
FV slope	−61.89 ± 14.45	−67.16 ± 11.31 *	−0.36	−62.91 ± 11.89	−54.05 ± 9.98 **	0.745	0.378	0.002
RFpeak (%)	49.75 ± 5.26	53.50 ± 4.01 ***	0.71	51.18 ± 3.46	49.36 ± 4.48 *	−0.526	0.173	0.039

Note: Within-group significance: * *p* < 0.05, ** *p* < 0.01, *** *p* < 0.001. ES = Cohen’s d.

## Data Availability

The data presented in this study are available on request from the corresponding author.

## References

[1] Faude O., Koch T., Meyer T. (2012). Straight sprinting is the most frequent action in goal situations in professional football. J. Sports Sci..

[2] Longo U.G., Sofi F., Candela V., Risi Ambrogioni L., Pagliai G., Massaroni C., Schena E., Cimmino M., D’aNcona F., Denaro V. (2021). The influence of athletic performance on the highest positions of the final ranking during 2017/2018 Serie A season. BMC Sports Sci. Med. Rehabil..

[3] Palucci Vieira L.H., Carling C., Barbieri F.A., Aquino R., Santiago P.R.P. (2019). Match Running Performance in Young Soccer Players: A Systematic Review. Sports Med..

[4] Fernández-Galván L., Boullosa D., Jiménez-Reyes P., Cuadrado-Peñafiel V., Casado A. (2021). Examination of the Sprinting and Jumping Force-Velocity Profiles in Young Soccer Players at Different Maturational Stages. Int. J. Environ. Res. Public Health.

[5] Fernández-Galván L., Jiménez-Reyes P., Cuadrado-Peñafiel V., Casado A. (2022). Sprint Performance and Mechanical Force-Velocity Profile among Different Maturational Stages in Young Soccer Players. Int. J. Environ. Res. Public Health.

[6] Nicholson B., Dinsdale A., Jones B., Till K. (2021). The Training of Short Distance Sprint Performance in Football Code Athletes: A Systematic Review and Meta-Analysis. Sports Med..

[7] Morin J.B., Samozino P. (2016). Interpreting power-force-velocity profiles for individualized and specific training. Int. J. Sports Physiol. Perform..

[8] Samozino P., Rabita G., Dorel S., Slawinski J., Peyrot N., de Villarreal E.S., Morin J.-B. (2016). A simple method for measuring power, force, velocity properties and mechanical effectiveness in sprint running. Scand. J. Med. Sci. Sports..

[9] Alcaraz P.E., Carlos-Vivas J., Oponjuru B.O., Martínez-Rodríguez A. (2018). The Effectiveness of Resisted Sled Training (RST) for Sprint Performance: A Systematic Review and Meta-analysis. Sports Med..

[10] Mainer-Pardos E., Khalili S., Villanueva-Guerrero O., Clemente F., Nobari H. (2024). The effects of resisted sprint training programs on vertical jump, linear sprint and change of direction speed in male soccer players: A systematic review and meta-analysis. Acta Kinesiol..

[11] Petrakos G., Morin J., Egan B. (2016). Resisted Sled Sprint Training to Improve Sprint Performance: A Systematic Review. Sports Med..

[12] Bachero-Mena B., González-Badillo J. (2014). Effects of Resisted Sprint Training on Acceleration With Three Different Loads Accounting for 5, 12.5, and 20% of Body Mass. J. Strength Cond. Res..

[13] Lahti J., Huuhka T., Romero V., Bezodis I., Morin J.B., Häkkinen K. (2020). Changes in sprint performance and sagittal plane kinematics after heavy resisted sprint training in professional soccer players. PeerJ.

[14] Zafeiridis A., Saraslanidis P., Manou V., Ioakimidis P., Dipla K., Kellis S. (2005). The effects of resisted sled-pulling sprint training on acceleration and maximum speed performance. J. Sports Med. Phys. Fit..

[15] Behm D.G., Young J.D., Whitten J.H.D., Reid J.C., Quigley P.J., Low J., Li Y., Lima C.D., Hodgson D.D., Chaouachi A. (2017). Effectiveness of Traditional Strength vs. Power Training on Muscle Strength, Power and Speed with Youth: A Systematic Review and Meta-Analysis. Front. Physiol..

[16] Martín-Moya R., Silva A.F., Clemente F.M., González Fernández F.T. (2023). Effects of combined plyometric, strength and running technique training program on change-of-direction and countermovement jump: A two-armed parallel study design on young soccer players. Gait Posture.

[17] Ramirez-Campillo R., Castillo D., Raya-González J., Moran J., De Villarreal E., Lloyd R. (2020). Effects of Plyometric Jump Training on Jump and Sprint Performance in Young Male Soccer Players: A Systematic Review and Meta-analysis. Sports Med..

[18] Styles W.J., Matthews M.J., Comfort P. (2016). Effects of strength training on squat and sprint performance in soccer players. J. Strength Cond. Res..

[19] Morin J., Edouard P., Samozino P. (2011). Technical ability of force application as a determinant factor of sprint performance. Med. Sci. Sports Exerc..

[20] Baena-Raya A., García-Mateo P., García-Ramos A., Rodríguez-Pérez M., Soriano-Maldonado A. (2021). Delineating the potential of the vertical and horizontal force-velocity profile for optimizing sport performance: A systematic review. J. Sports Sci..

[21] Cross M.R., Lahti J., Brown S.R., Chedati M., Jiménez-Reyes P., Samozino P., Eriksrud O., Morin J.-B. (2018). Training at maximal power in resisted sprinting: Optimal load determination methodology and pilot results in team sport athletes. PLoS ONE.

[22] Rodriguez-Rosell D., Sáez de Villarreal E., Mora-Custodio R., Asian-Clemente J. (2020). Effects of Different Loading Conditions During Resisted Sprint Training on Sprint Performance. J. Strength Cond. Res..

[23] Derakhti M., Bremec D., Kambič T., Siethoff L., Psilander N. (2021). Four Weeks of Power Optimized Sprint Training Improves Sprint Performance in Adolescent Soccer Players. Int. J. Sports Physiol. Perform..

[24] Niederdraeing L., Zentgraf K. (2025). Force-Velocity Profiling among Different Maturational Stages in Young Soccer Players: A Cross-Sectional Study. Int. J. Strength Cond..

[25] Bhide A., Shah P., Acharya G. (2018). A simplified guide to randomized controlled trials. Acta Obstet. Gynecol. Scand..

[26] Miola A., Espósito A., Miot H. (2024). Techniques for randomization and allocation for clinical trials. J. Vasc. Bras..

[27] McKay A.K.A., Stellingwerff T., Smith E.S., Martin D.T., Mujika I., Goosey-Tolfrey V.L., Sheppard J., Burke L.M. (2022). Defining training and performance caliber: A participant classification framework. Int. J. Sports Physiol. Perform..

[28] Lakens D. (2022). Sample Size Justification. Collabra Psychol..

[29] Orzechowski M., Wozniak K., Timmermann C., Steger F. (2021). Normative framework of informed consent in clinical research in Germany, Poland, and Russia. BMC Med. Ethics.

[30] Runacres A., Mackintosh K., Mcnarry M. (2023). Investigating the kinetics of repeated sprint ability in national level adolescent hockey players. J. Sports Sci..

[31] Moseley A., LeBlanc M., Freidlin B., Shallis R.M., Zeidan A.M., Sallman D.A., Erba H.P., Little R.F., Othus M. (2025). Evaluating the impact of stratification on the power and cross-arm balance of randomized phase 2 clinical trials. Clin. Trial.

[32] Blanca M., Arnau J., García-Castro F., Alarcón R., Bono R., Espejo B. (2023). Repeated measures ANOVA and adjusted F-tests when sphericity is violated: Which procedure is best?. Front. Psychol..

[33] Kinney A., Eakman A., Graham J. (2020). Novel Effect Size Interpretation Guidelines and an Evaluation of Statistical Power in Rehabilitation Research. Arch. Phys. Med. Rehabil..

[34] Sudlow A., Galantine P., Del Sordo G., Raymond J.-J., Dalleau G., Peyrot N., Duché P. (2025). Effects of maximal power and the force–velocity profile on sprint acceleration performance according to maturity status and sex. J. Sports Sci..

[35] Morin J., Petrakos G., Jiménez-Reyes P., Brown S., Samozino P., Cross M. (2017). Very-Heavy Sled Training for Improving Horizontal-Force Output in Soccer Players. Int. J. Sports Physiol. Perform..

[36] Pantoja P., Carvalho A., Ribas L., Peyré-Tartaruga L. (2018). Effect of weighted sled towing on sprinting effectiveness, power and force-velocity relationship. PLoS ONE.

[37] Stavridis I., Ekizos A., Zisi M., Agilara G.-O., Tsolakis C., Terzis G., Paradisis G. (2023). The Effects of Heavy Resisted Sled Pulling on Sprint Mechanics and Spatiotemporal Parameters. J. Strength Cond. Res..

[38] Amore M., Minciacchi D., Panconi G., Guarducci S., Bravi R., Sorgente V. (2024). Impact of Sled-Integrated Resisted Sprint Training on Sprint and Vertical Jump Performance in Young U-14 Male Football Players. J. Funct. Morphol. Kinesiol..

[39] Helland C., Haugen T., Rakovic E., Eriksrud O., Seynnes O., Mero A., Paulsen G. (2019). Force–velocity profiling of sprinting athletes: Single-run vs. multiple-run methods. Eur. J. Appl. Physiol..

[40] Sugisaki N., Tsuchie H., Takai Y., Kobayashi K., Yoshimoto T., Kanehisa H. (2025). Causes of Different Force–Velocity Relationships in Sprint Running Depending on Horizontal Loads and Profiling Methods. Med. Sci. Sports Exerc..

